# Effect of dimethyl carbonate addition on ethanol–gasoline fuel blend

**DOI:** 10.1038/s41598-023-41329-5

**Published:** 2023-09-04

**Authors:** Manal Amine, Y. Barakat

**Affiliations:** Processes Design & Develop Department, Egyptian Petroleum Research Institute, Cairo, Egypt

**Keywords:** Chemistry, Energy science and technology

## Abstract

The growing need for renewable and environmentally friendly sources of energy has motivated a lot of researchers to direct their efforts to meet these challenges. The use of renewable additives to gasoline, such as ethanol and methanol, has been a successful solution. However, blending ethanol into gasoline has some drawbacks, including increased gasoline volatility and significant changes in the distillation curve. This study investigated the effects of blending the eco-friendly dimethyl carbonate (DMC) with various concentrations into ethanol-gasoline fuel blend (E10) on some volatility parameters and octane number, which have not been previously reported in the literature. The fuel samples were formulated by mixing E10 with (0.0%, 2.0%, 4.0%, 6.0%, 8.0%, and 10.0%) of dimethyl carbonate. The main properties of the fuel samples were measured such as distillation curve, and octane number. The distillation process was carried out in accordance with ASTM-D86 while vapor pressure was measured in accordance with ASTM-D5191. The obtained results revealed interesting outcomes that may spark the interest of refineries in this promising fuel additive. Addition of DMC to gasoline-ethanol blend was found to have insignificant impact on the volatility of fuel. The results demonstrate that addition of ethanol to gasoline causes a significant decrease in T50 by about 20 °C, while addition of 10 volume percent of DMC to E10 causes an increase in T50 by about 2 °C. The obtained results showed also that the addition of 10 vol% of DMC to E10 fuel blend considerably increases the RON and MON by about 4 and 3.5 points, respectively.

## Introduction

The growing need for renewable and environmentally friendly energy sources has motivated a lot of researchers to direct their efforts to meet these challenges. Transportation section is one of the most sources that participate in climate change. Transport accounted for more than the quarter of greenhouse gases in 2020 in USA, which is the largest participation in the greenhouse gas (GHG) emissions and climate change^1^. Most of the transportation vehicles run on gasoline and diesel fuel, which contribute significantly to increased air pollution when combustion^2^. Oxygenated compounds were first used as environmentally friendly alternatives to the hazardous tetra ethyl lead compounds which were used to boost the octane number of gasoline. Ethanol and Methyl-tert butyl ether (MTBE) represented the most common oxygenates used to enhance the quality of gasoline as they have high-octane number^3^. They have been used in the United States 1980s. According to the literature, both ethanol and MTBE have been found to decrease exhaust emissions of carbon monoxide (CO) by 18% and 36%, respectively^4^. Methyl-tert butyl ether (MTBE) was the most common used oxygenated additive used to enhance the octane number of gasoline^3,5^. For health repercussions, the use of MTBE as gasoline additive has been banned because it has been found to contaminate the groundwater^6^. Short chain alcohols have been used as alternatives to MTBE. Ethanol is the most common oxygenate as it is an environmentally friendly and renewable source of energy. Addition of ethanol to gasoline has several advantages for example; it improves the octane number, it contributes significantly to the improvement of the engine performance and improves the combustion process and thus improves exhaust emission^7–13^. The higher octane number of ethanol indicates a relatively high antiknock value, good properties, and quality at high compression ratio, thereby avoiding self-ignition at end-gas region^14,15^. Dimethyl carbonate (DMC) is also an environmentally friendly oxygenated solvent. It is produced industrially by the carbonylation processes in which the CO_2_ gas is used as a raw material to produce DMC. DMC is prepared by a simple and cheap method which involves a direct reaction of carbon dioxide and methanol. So its production contributes in decreasing greenhouse gases and thus contributes in improving air quality^16,17^. DMC was investigated as oxygenated additive to gasoline and diesel and the results obtained were promising in improving tailpipe emissions and improving engine performance. The exhaust emitted gases HC, PM, and CO were found to be reduced when DMC was blended with diesel or gasoline fuel. The reduction was attributed by the researchers to the improvement in oxygen content resulting from blending DMC with the fuel^18–23^.

Ethanol-gasoline blends used as automotive fuel is a potential way for extending the fuel reserves. However many of serious problems have been detected with the use of a high concentration of ethanol in excess of 10 volume percent in gasoline fuel. A high concentration of ethanol may cause degraded engine performance such as poor cold start, hesitation, rough idling or engine stall^24–26^. Also, high concentrations of ethanol may cause valves wearing and elastomers deterioration^27–29^. High concentration of ethanol can also cause a decreasing in the fuel quality due to the phase separation that could be happen at high humidity and low temperature. Ethanol is highly hygroscopic so it absorbs the ambient water and separates at the bottom of the fuel^24,30,31^. This phase separation may also lead to corrosion to the metallic parts in the engine or in the fuel tanks^32^. Another drawback of using high concentration of ethanol in gasoline is that it dramatically affects the distillation curves and the volatility criteria of the fuel blend which negatively affects engine performance^33^. As a result, the acceptable limit for ethanol in gasoline approved by EPA is 10 volume percent. Gasoline containing 10 volume percentage of ethanol is only allowed for using in the current engines while higher concentrations of ethanol in gasoline require engine modifications.

Dual oxygenated gasoline formulation may meet the fuel requirements and be more suitable for the engine performance than using high concentration of single oxygenated fuel blends.

A lot of works discussed the effect of blending another oxygenated additive with ethanol gasoline formulations to avoid or diminishes the drawbacks of ethanol addition. Some researchers concluded that it is possible to design dual-alcohol formulations to have a vapor pressure very close to that of gasoline. They found that the use of higher alcohols such as iso-butanol and 3-methyl-3-pentanol as components in ethanol gasoline blends reduce the drawbacks associated with using ethanol alone in gasoline fuel. They found also that dual alcohol blends showed reasonable properties such as viscosity, volatility and water tolerance^34^.

Some works studied the impact of blending ethyl acetate on the properties of gasoline- ethanol formulations and the results showed that ethyl acetate increases the stability, the water tolerance, and the octane number of the fuel blends with no adverse effects on the volatility properties^35,36^. Amine and Barakat studied also the impact of the cyclohexanol on the water tolerance of ethanol-gasoline blends and they found that 3 volume percent of cyclohexanol increase the water tolerance of the 20% ethanol-gasoline blends by six times without any negative effect on the volatility properties of the fuel blends^37^.

In this work we aim at investigating the eco- friendly DMC to increase the gasoline reserve without negative impacts on the gasoline volatility by blending it with the acceptable percentage of ethanol (10 vol%) into gasoline. The presented work focuses on the impact of blending dimethyl carbonate, with various concentrations, into gasoline-ethanol blend (E10) on the distillation curve. The impact was illustrated through calculating the area under the distillation curve, which has not been presented in any previous similar studies.

## Materials and methods

### Fuel formulation

Cairo Petroleum Company provided us with the base gasoline, which was subjected to gas chromatographic analysis to determine its composition, as shown in Table 1. Dimethyl carbonate (99%) was procured from ACROS ORGANICS Co whereas ethanol (99.9%) was procured from Carlo Erpa Co. A set of fuel samples (E10, E10-2DMC, E10-4DMC, E10-6DMC, E10-8DMC, and E10-10DMC) in addition to the base gasoline (G) were formulated as indicated in Table 2. The main properties of the blend constituents are given in Table 3.

**Table 1:** Chemical composition of base gasoline.

Components	Wt%
Propane	0.033
i-Butane	0.422
n-Butane	2.080
i-Pentane	10.504
n-Pentane	9.932
Hexanes	28.726
Benzene	2.219
Heptanes	16.560
Toluene	8.176
Octane	8.712
Ethyl-benzene	0.812
p.m-Xylene	3.861
o-Xylene	1.140
Nonanes	3.472
Decanes	1.867
Undecanes	0.801
Dodecanes	0.369
Tridecanes	0.143
Tetradecanes	0.077
Pentadecanes	0.035
Hexadecanes	0.021
Heptadecanes	0.016
Octadecanes	0.012
Nonadecanes	0.007
Icosanes	0.003
Total	100.000
Total aromatics	16.208
Total paraffenes	83.792

**Table 2:** Composition and volatility parameters of fuel samples.

Blend composition vol%	G	E10	E10-2DMC	E10-4DMC	E10-6DMC	E10-8DMC	E10-10DMC
Gasoline	100	90	88	86	84	82	80
Ethanol	0	10	10	10	10	10	10
DMC	0	–	2	4	6	8	10
Total	100	100	100	100	100	100	100
Distillation data							
IBP, °C	41.06	42.04	42.37	43.38	42.27	40.05	42.05
T10, °C	58.46	52.65	52.72	53.33	53.16	53.16	53.78
T20, °C	64.39	56.13	56.25	56.91	56.79	56.62	56.64
T30, °C	70.69	59.39	59.49	59.94	59.98	59.81	60.19
T40, °C	77.75	62.63	62.47	63.09	63.02	63.16	63.41
T50, °C	85.75	66.06	67.11	67.7	66.67	67.16	67.96
T60, °C	95.02	87.9	87.75	84.16	79.84	79.25	78.95
T70, °C	105.87	102.23	101.51	100.12	96.86	93.67	92.95
T80, °C	120.63	116.96	116.47	117.25	115.2	112.05	110.95
T90, °C	146.46	143.17	143.27	145.45	142.52	136.84	137.38
T95, °C	181.45	178.96	179.99	189.78	180.99	161.98	163.23
FBP	202.25	194.38	196.44	198.7	195.39	182.91	192.29
VP (kPa)	58.1	67	67	65	65.4	64	-
E70	31	54	54	54	56	54	54
E100	68	71	71	73	74	76	76
E150	93	94	94	94	94	94	94

**Table 3:** Properties of the main constituents of the fuel blends.

Property	Gasoline^38^	Ethanol^39,40^	Dimethylcarbonate^17,20^
Molar mass, g/mol	–	46.07	90.08
Boiling Point, °C	(41–202)	78.4	90
Density, g/cm^3^	0.7200	0.789	1.069
Specific gravity	0.7207	0.789	1.06
Flash point, °C	< − 27	13–14	18
RON	84	109	111
Solubility in water	Insoluble	Soluble	13.9 g/100 ml

### Characterization of fuel samples

The fuel blends were distilled by using STARDist Automatic Distillation unit MODEL No.913021 according to ASTM-D86. The vapor pressure was determined using the SETAvap II Automatic Vapor Pressure Tester-81000-2 according to ASTM-D5191. ZELTEX ZX-101X portable near-infrared octane-cetane analyzer was used to measure the octane rating. The area under the distillation curve was calculated by definite integrals^41^. Table 4 displays the uncertainty of the measured parameters.

**Table 4:** Uncertainty of the parameters.

Vapor pressure	± 0.5 kPa
Distilled fuel	± 0.01 ml
RON	± 0.25
MON	± 0.12

## Results and discussion

### Volatility

Volatility is a very important parameter in the fuel industry. The fuel ignites in the vapor state in the engine so it converts to vapor first. The Volatility of gasoline is must adjusted to ensure good startability and driveability. It is also crucial to be controlled for environmental concerns, and there are numerous parameters used to estimate the volatility of the fuel.

#### Distillation curve

One of the most common and important parameters of gasoline industry is the distillation curve due to its importance for the engine performance. It establishes a relationship between the volume of the fuel recovered and the temperature of distillation. It presents important data that assess the gasoline volatility. T10, T50, and T90 are the temperature degrees at which 10, 50, and 90 volume percentages of the fuel distilled. From these data several fuel parameters can be deduced. ASTM-D4814 sets limits for these temperature degrees to control the gasoline volatility as given in Table 5. EN − 228 uses another data deduced from the distillation curve which are E70, E100, and E150.

**Table 5:** Standards of the volatility parameters.

Parameter (units)	Standard	Minimum	Maximum
RVP (kPa)	ASTM 4814	–	79
T10, °C	ASTM 4814	–	60
T50, °C	ASTM 4814	77	116
T90, °C	ASTM 4814	–	185
RVP (kPa)	EN228	50	80
E70% (v/v)	EN228	22	50
E100% (v/v)	EN228	46	71
E150% (v/v)	EN228	75	-

**Figure 1:**
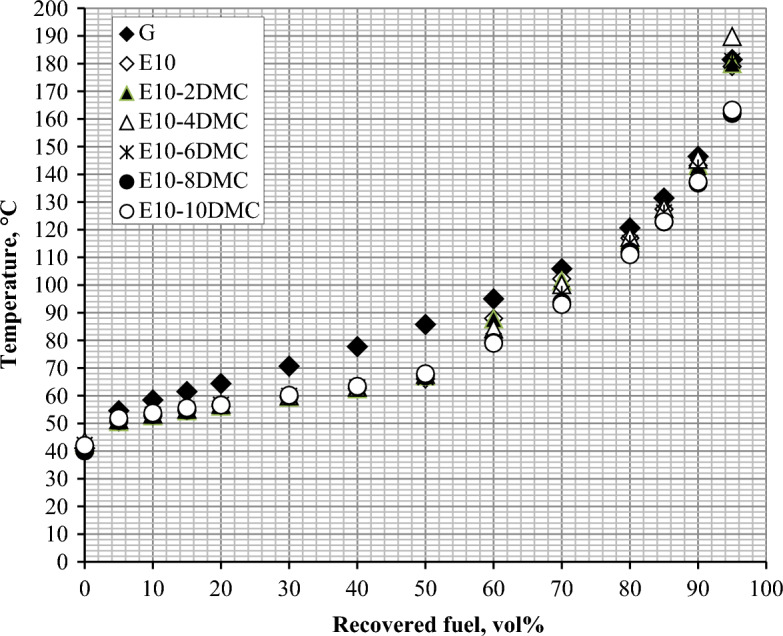
The impact of blending DMC into gasoline-ethanol blends on the distillation curve.

**Figure 2:**
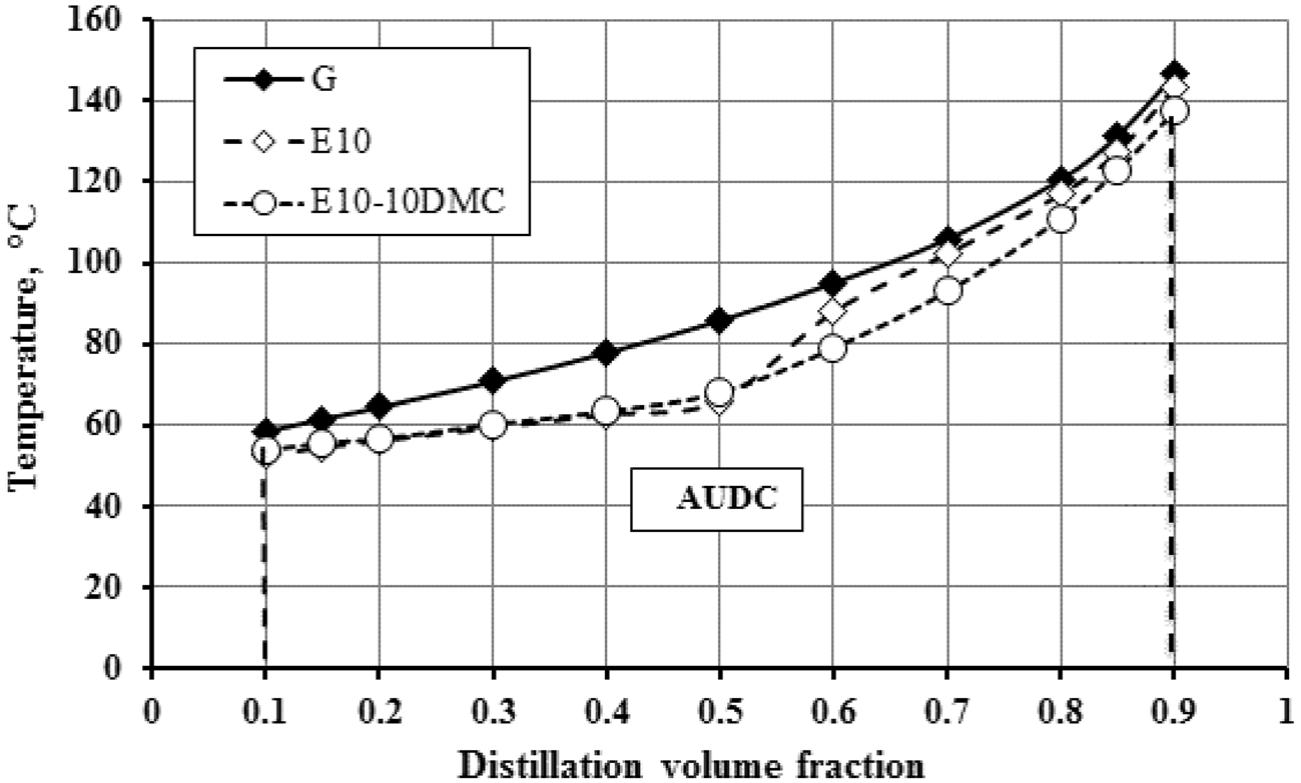
Displaying the area under distillation curve, the effect of ethanol on distillation curve of gasoline fuel, and the effect of blending 10% DMC on distillation curve of gasoline-ethanol blend.

**Figure 3:**
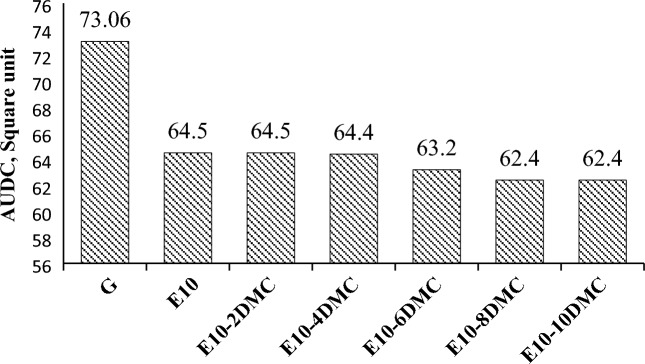
Impact of ethanol on the area under distillation curve of gasoline and the impact of DMC on the area under distillation curve of gasoline-ethanol blends.

Addition of ethanol to gasoline dramatically changes the distillation curve as shown in Figs. 1 and 2. This change was attributed to the azeotrope formation that happen between ethanol and some hydrocarbons present in gasoline. This azeotropic mixture possesses lower boiling range so the distillation curve shifts downwards in case of adding ethanol to gasoline^42^. In the present work we investigate the effect of dual oxygenates on the distillation curve of gasoline. Figure 1 shows that blending DMC into gasoline-ethanol blends does not change the front end of the distillation curve. Looking at the front end (up to 50% of fuel distilled) of the distillation curves in Fig. 1, we notice that the curves are overlapping on each other, which suggest that the addition of DMC has minimal influence on the lighter fraction of gasoline. The change in the curves is beginning from 60% of fuel distilled; we found that the curves shift slightly downwards as the amount of DMC in the fuel sample increases as clearly shown in Fig. 1. This may be attributed to the formation of azeotropic mixture between DMC and higher fraction of gasoline. This azeotropic mixture has boiling range slightly lower than boiling range of gasoline. For more clarification and comparing the effect of duel oxygenates on the distillation curve of gasoline; the area under each distillation curve is estimated as indicated in Methods section. Figure 2 shows the area under the distillation curve and shows the changes made to the distillation curve of gasoline by adding ethanol and the changes made to the distillation curve of E10 by adding DMC. Figure 3 describes the extent to which the area under the curve in E10 blend is significantly decreased. It is decreased from 73.1 to 64.5 square units (about 9 square unit) while the addition of DMC causes insignificant decrease in the area under the curves of gasoline-ethanol blends. For blend E10-10DMC, the area decreases only by about 2 square units as declared in Table 6. These results may be attributed to the formation of an azeotropic mixture between ethanol and the light fraction of gasoline, which has a boiling range much lower than that of gasoline. Thus, the distillation curve shifts downward, causing a significant decrease in the area under the curve. On the other hand, in the case of DMC, we found that the azeotrope formed between DMC and higher fraction of gasoline has a boiling range slightly lower than that of gasoline, as indicated by the distillation data. Therefore, the curve shifts slightly downward, causing an insignificant decrease in the area under the curve.

**Table 6:** The derived trend line equations of the distillation curve from 10 to 90% distilled fuel.

Sample	Polynomial equation	R^2^	AUDC, square unit
G	y = 102.14^2^ – 1.1871x + 59.756	0.9928	73.1
E10	y = 161.14x^2^ – 53.72x + 58.539	0.9926	64.5
E10-2DMC	y = 160.97x^2^ – 53.528x + 58.625	0.9937	64.5
E10-4DMC	y = 174.15x^2^ – 67.08x + 61.245	0.9947	64.4
E10-6DMC	y = 177.77x^2^ – 74.514x + 62.365	0.9939	63.2
E10-8DMC	y = 162x^2^ – 64.636x + 61.131	0.9948	62.4
E10-10DMC	y = 164.2x^2^ – 67.879x + 62.161	0.9939	62.4

##### T10, T50, and T90

**Figure 4:**
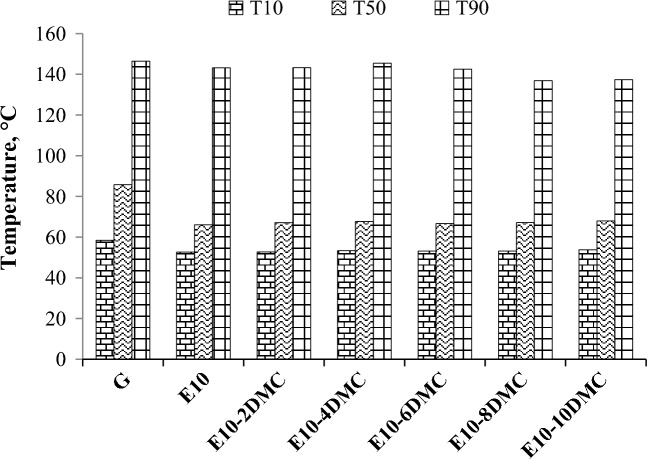
The impact of ethanol on the T10, T50, and T190 of gasoline and the impact of DMC on the T10, T50, and T190 of gasoline-ethanol blends.

These temperature degrees are controlled by ASTM-D4814 as they are very important for engine performance. T10 is the temperature at which 10% of the fuel is distilled. Its importance is only to ensure a good cold start especially in cold weather and to ensure no increase in the emissions of the volatile organic compounds (VOCs). T50 characterizes the midrange volatility which ensures the engine warming-up, acceleration and avoid ice formation. Controlling T90 is significant for fuel economy^43^. Figure 4 shows the impact of the duel blends on these temperature degrees. The decrease in T10 was insignificant compared to the decrease in T50 of E10 fuel blend. For E10 fuel blend, T50 was decreased by about 20 °C. These results can be attributed to the fact that ethanol can form azeotropic mixture with the light fractions of gasoline causing the decrease in the temperature that happened in the front end of the distillation curve. On the contrary, blending DMC in E10 fuel blend has no effect on the reduction that occurred for T10 and T50 as shown in Fig. 4. This could be explained by the non-formation of an azeotropic mixture between DMC and the lighter components of gasoline. So we can conclude that the decrease in the front end and midrange temperature degrees of the studied blends is attributed to the presence of ethanol and not the presence of DMC^42^. T90 of gasoline was slightly reduced when ethanol was added by about 2% while it decreased by about 6% for E10-10DMC. This results confirm the assumption that ethanol does not form azeotropic mixture with the higher fraction of gasoline, while DMC does form one with the the higher fraction of gasoline.

##### E70, E100, and E150

**Figure 5:**
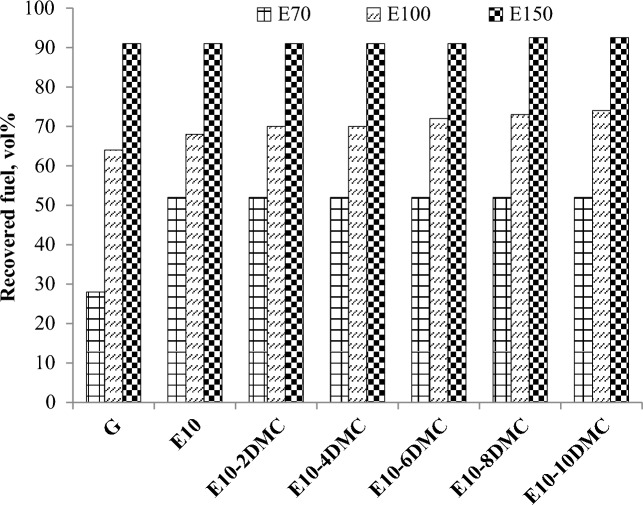
The impact of ethanol on the E70, E100, and E150 of gasoline and the impact of DMC on the E70, E100, and E150 of gasoline-ethanol blends.

The European Standards for gasoline En 228 set some parameters controlling the distillation curve of the fuel such as E70, E100 and E150 which are the volume percentages of the fuel distilled at temperature degrees 70, 100, and 150 °C; respectively. Figure 5 shows the impact of the dual oxygenated blend on E70, E100, and E150. The figure shows that the recovery of the fuel was increased from 28 to 52 vol% (i.e. by about 24%) for E10 fuel blend at 70 °C while the addition of DMC to E10 does not almost cause any increase in E70. E100 was slightly increased for E10 blend from 64 to 68 vol% (i.e. by about 4%) while it increased by addition of high concentrations of DMC to E10 fuel blend to reach 10% by the addition of 10 volume percentages of DMC to E10 fuel blend. For E150, there is no significant change for almost all the fuel blends. It was found that E150 for E10 increased by 0.0%, while that of E10-10DMC increased by 1.6%. This results confirm the assumption that ethanol does not form an azeotropic mixture with the higher fraction of gasoline, while DMC does form one with slightly lower boiling range than gasoline. From the previous results we can add DMC to E10 fuel blends up to 10 vol% without fearing of increasing the volatility of the fuel blends.

### Octane rating and Knock resistance

Knocking resistance is the capability of the gasoline not to ignite spontaneously under the effect of the high compression. Octane rating is an important parameter for the efficiency and the performance of the engine. There are two methods for assessment the octane rating of gasoline. One of them is to test the fuel sample at mild conditions of temperature, speed and load which is the common number in gasoline market and known as the Research octane number (RON). The second method measures the fuel sample at severe conditions of temperature, load and speed which is known as the Motor octane number (MON). The average of RON and MON is called the antiknock index (AKI). The octane rating depends on the gasoline composition^5^. Straight run gasoline which is rich in paraffinic hydrocarbons usually has octane number of about 70. Gasoline rich in aromatics and isomerate (highly branched compounds) is of higher octane number than the straight run gasoline. However, gasoline must be formulated in such a way as to ensure a reasonable octane number and meet environmental concerns by setting limits to the aromatic contents and isomerate fraction, which increases the emission of volatile organic compounds.

**Figure 6:**
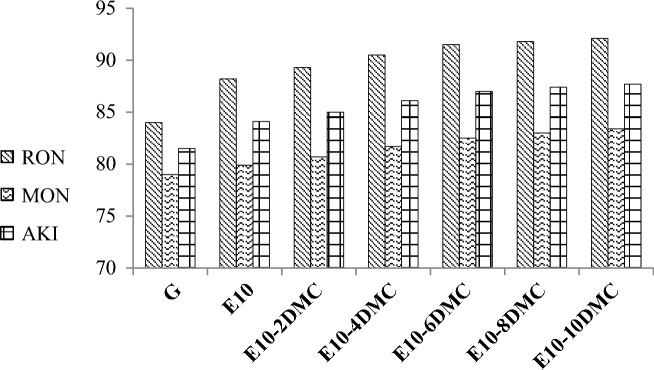
The impact of ethanol on the RON, MON, and AKI of gasoline and the impact of DMC on the RON, MON, and AKI of gasoline-ethanol blends.

Figure 6 shows how the addition of DMC participates in increasing the octane rating of E10 fuel blends. The RON increased for E10-10DMC blend by about 4 points which is higher than the effect of the addition of 10% of ethyl-tertbutyl ether (ETBE) and methyl tert-butyl ether (MTBE) to gasoline^44^ while its effect on RON is to some extent similar to that of ethanol^8^. The improvement of octane rating leads to enhancing the engine efficiency^11^. The Figure shows also that 10% of DMC considerably increases the MON of E10 fuel blend by about 3.5. AKI was found to be also increased by about 3.6 points. From these results, we can assume that DMC can improve the engine efficiency which has already been proven by literature.

## Conclusion

The results of the experiments allow us to draw the following conclusions: the values of the volatility criteria of E10 fuel blends containing DMC have almost not changed from those of the E10 blend. Also, it was found that blending DMC into the E10 fuel blend significantly improves the octane rating. The addition of 10 volume percent of DMC increases the RON by about 4 points. DMC could be a promising environmentally friendly octane booster additive for E10 fuel blend while avoiding the drawbacks caused by using higher concentrations of ethanol. The authors suggest further investigations to evaluate DMC well as an additive to gasoline- ethanol blend, including physicochemical properties, fuel consumption and engine performance.

## Findings and practical implication

In this section, we highlight the most important findings, which indicate that the addition of dimethyl carbonate (DMC) has little effect on the volatility properties of gasoline blended with ethanol. Also, it increases the octane number of the fuel blends, which may improve the fuel performance in the engine. These results can increase the possibility of blending DMC with ethanol-gasoline blends instead of using high concentrations of ethanol alone, which has negative effects on some gasoline properties that require engine modifications. Therefore, we strongly recommend conducting more research on such blends, such as performance and consumption experiments, as well as exhaust emissions.

## Data Availability

All data generated or analyzed during this study are included in this published article.

## Abbreviations

AKI: Antiknock index
ASTM: American society for testing and materials
CO: Carbon monoxide
CO2: Carbon dioxide
DMC: Dimethyl carbonate
E10: Gasoline fuel containing 10 volume percent of absolute ethanol
E100: Evaporated fuel at 100 °C
E150: Evaporated fuel at 150 °C
E70: Evaporated fuel at 70 °C
EN: European standards
EPA: Environmental protection agency
ETBE: Ethyl-tert-butyl ether
G: Base gasoline
E10-10DMC: Gasoline blend containing 10 vol% ethanol and 10 vol% DMC
E10-2DMC: Gasoline blend containing 10 vol% ethanol and 2 vol% DMC
E10-4DMC: Gasoline blend containing 10 vol% ethanol and 4 vol% DMC
E10-6DMC: Gasoline blend containing 10 vol% ethanol and 6 vol% DMC
E10-8DMC: Gasoline blend containing 10 vol% ethanol and 8 vol% DMC
GHG: Greenhouse gases
HC: Hydrocarbon emission
MON: Motor octane number
MTBE: Methyl-tert-butyl ether
PM: Particulate matter
RON: Research octane number
T10: Temperature at which 10% of fuel distilled
T50: Temperature at which 50% of fuel distilled
T90: Temperature at which 90% of fuel distilled
